# Radiation-induced angiosarcoma of the brain

**DOI:** 10.1259/bjrcr.20150374

**Published:** 2015-12-01

**Authors:** Sodai Hoshiai, Tomohiko Masumoto, Masahide Matsuda, Narushi Sugii, Noriaki Sakamoto, Manabu Minami

**Affiliations:** ^1^ Department of Diagnostic and Interventional Radiology, University of Tsukuba Hospital, Ibaraki, Japan; ^2^ Department of Neurosurgery, University of Tsukuba Hospital, Ibaraki, Japan; ^3^ Department of Diagnostic Pathology, University of Tsukuba Hospital, Ibaraki, Japan

## Abstract

Primary angiosarcoma of the central nervous systemis unusual.We encountered a case of radiation-induced angiosarcoma of the brain. A 65-year-old male was referred to our hospital with drowsiness for the last 6 months. He had undergone radiation therapy for pituitary adenoma 43 years ago. An MRI revealed a right temporal lobe tumour that consisted of a well-demarcated haemorrhagic lesion and an avid contrast enhancement, with significant vasogenic oedema. Surgical resection was performed and a post-operative pathological diagnosis of an angiosarcoma was made. A Thorotrast-associated angiosarcoma has been, hitherto, the only reported case of radiation-induced angiosarcoma of the brain. We present an extremely rare case of primary angiosarcoma of the brain, occurring after external beam radiotherapy.

## Summary

Angiosarcoma is a unusual malignant neoplasm of the vascular endothelium that occurs mainly in the skin and soft tissues.^1^ An angiosarcoma arising from the central nervous system (CNS) is extremely rare.^1,2^ Only one case of Thorotrast-associated angiosarcoma has ever been reported as a radiation-induced angiosarcoma of the brain.^2^ We encountered a case of angiosarcoma of the brain occurring 43 years after external beam radiotherapy of the patient.

## Case

A 65-year-old male was referred to our hospital. He had presented to another hospital 6 months earlier with drowsiness and a 54-mm diameter mass had been observed in the right temporal lobe on non-contrast CT scan. The patient had undergone surgery and cobalt-60-based external beam radiotherapy for pituitary adenoma 43 years ago. Parallel–opposed lateral radiotherapy targeting the pituitary lesion was employed with a total dose of 50 Gy in 25 fractions. The patient had recovered without residual symptoms but required hormone replacement for panhypopituitarism. He had also developed pontine infarction at 57 years of age. The results of physical and neurological examinations at the time of admission were normal. Laboratory evaluations of complete and differential blood counts, and serum chemistry were also normal.

**Figure 1.**
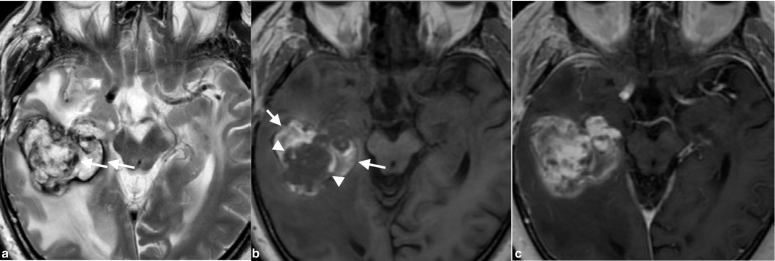
A well-demarcated mass is seen in the right temporal lobe with extensive vasogenic oedema on *T*_2_ weighted MRI. The mass shows hyper- and hypo-intensity, indicating haemosiderin deposition (arrows; a). *T*_1_ weighted image shows a hypointense mass (arrowheads) with hyperintense areas in the periphery, suggesting haemorrhagic foci (arrows; b). Contrast-enhanced *T*_1_ weighted image of the brain shows significant contrast enhancement in the hypointense areas on the pre-enhanced *T*_1_ weighted image (c).

**Figure 2.**
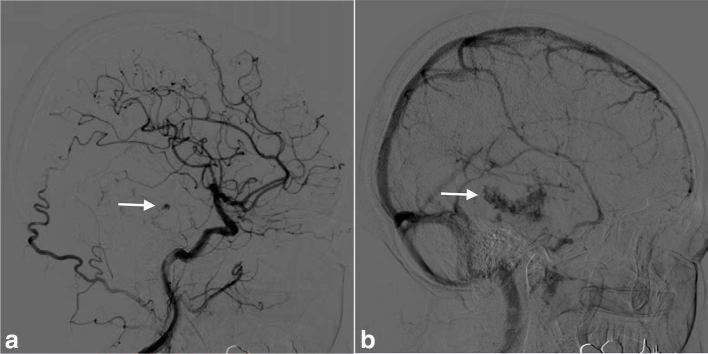
Right lateral common carotid angiogram of the brain shows contrast medium enhancement (arrow), supplied by the inferior branch of the right middle cerebral artery, in the arterial phase (a). The contrast medium pooling continues until the venous phase, suggesting the presence of a blood sinus-like structure in the mass (arrow; b).

**Figure 3.**
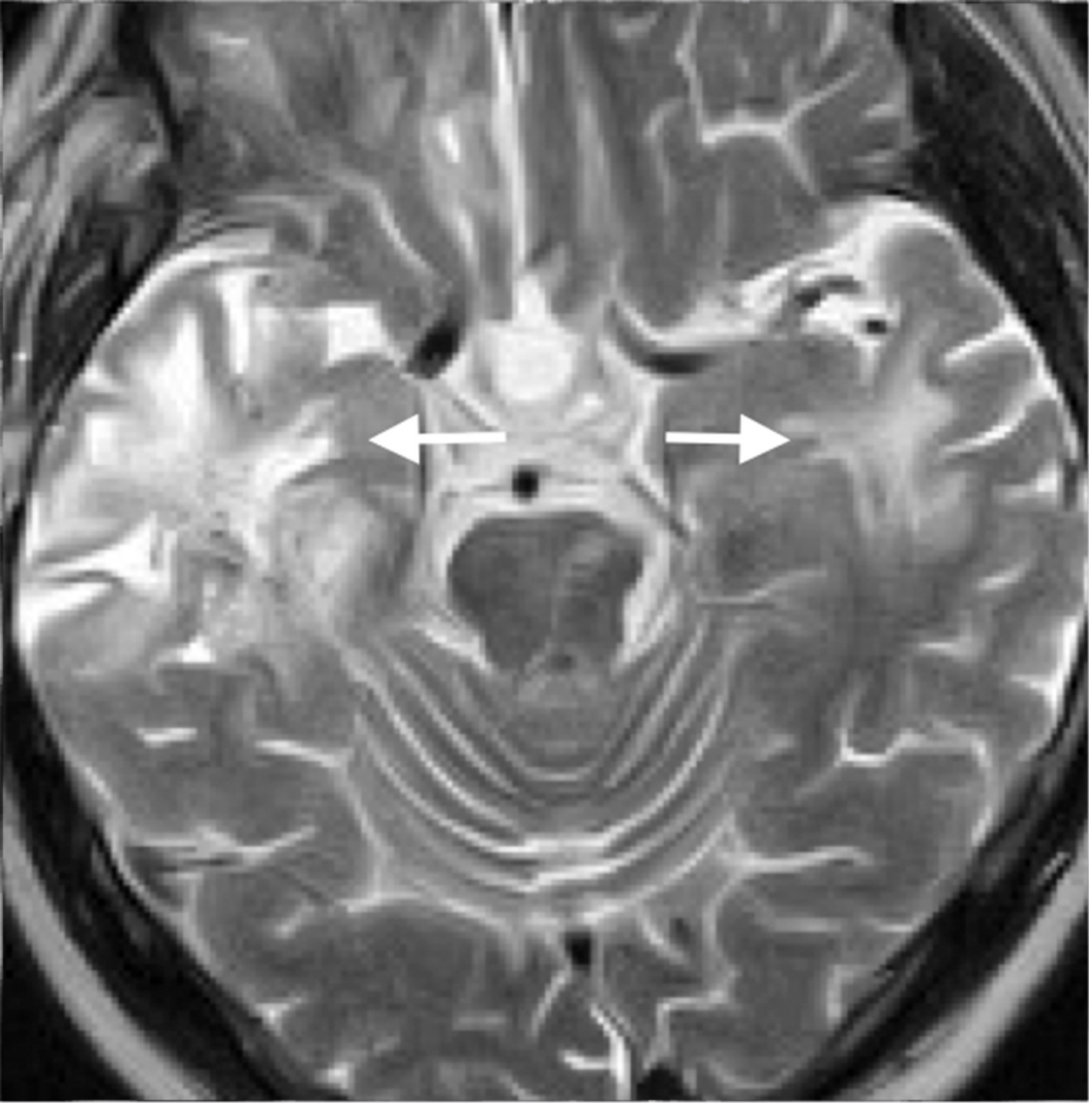
An MRI performed 1 year ago shows hyperintense areas in the bilateral temporal lobes, indicating radiation-induced change on a *T*_2_ weighted image (arrows).

The patient underwent a contrast-enhanced brain MRI that revealed a well-demarcated mass in the right temporal lobe (Figure 1). A mass consisting of hyper- and hypo-intensities was observed on *T*_2_ weighted imaging. There was extensive vasogenic oedema around the mass. The *T*_1_ weighted image showed a hypointense mass with hyperintense areas in the periphery, suggesting haemorrhagic foci. Contrast-enhanced *T*_1_ weighted image of the brain showed significant contrast enhancement in the hypointense area on the pre-enhanced *T*_1_ weighted image. The mass was supplied by the inferior branch of the right middle cerebral artery, and conventional angiography showed spotty stains in the arterial phase and spreading and pooling of the contrast medium in the venous phase, which suggested the presence of blood sinus-like structure in the mass (Figure 2). CT images of the chest, abdomen and pelvis were normal (data not shown). Because hyperintense areas in the bilateral temporal lobes, indicating radiation-induced changes, had been detected on the *T*_2_ weighted image 1 year before (Figure 3), the mass was considered to arise within the radiation field of the previous radiotherapy, which probably involved opposing portal irradiation. The preoperative diagnosis was radiation-induced lesions, including cavernous malformation and glioma. Surgical resection of the mass was performed and it was found to be highly haemorrhagic and richly vascularized. The mass and the hippocampus were resected because the mass bled easily and had invaded the hippocampus.

**Figure 4.**
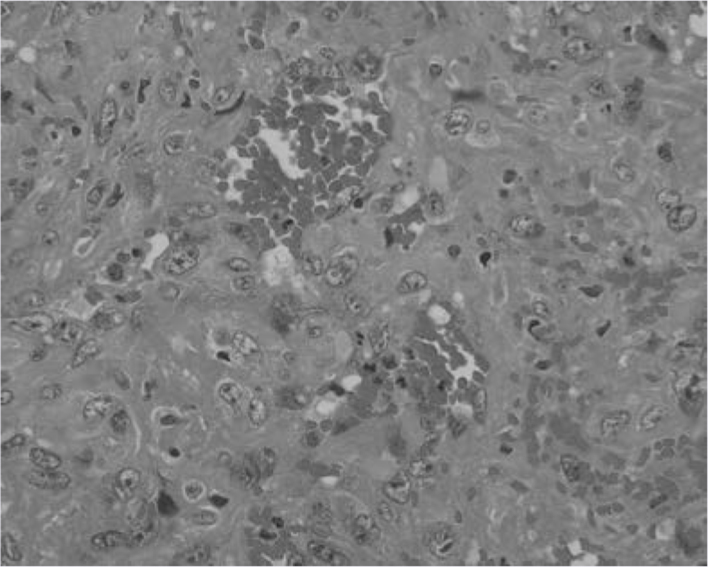
Haematoxylin and eosin-stained section of the temporal lobe mass (original magnification, ×400) reveals widespread haemorrhage and abnormal vessel proliferation in the tumour. Abnormal vessels contain organized blood clots, and have thick and hyalinized walls. The dilated intravascular lumens are covered by epithelial cells. These cells exhibit variable cytologic atypia and mitotic activity.

Pathologically, the mass exhibited widespread haemorrhages and abnormal vessel proliferation. The abnormal vessels contained organized blood clots, and had thick and hyalinized walls. The dilated intravascular lumens were covered by epithelial cells that were immunohistochemically positive for CD34. These cells exhibited variable cytologic atypia and mitotic activity (1–2  per 10 high power field) (Figure 4). These pathological findings were consistent with that of an angiosarcoma of the brain.^3^

**Figure 5.**
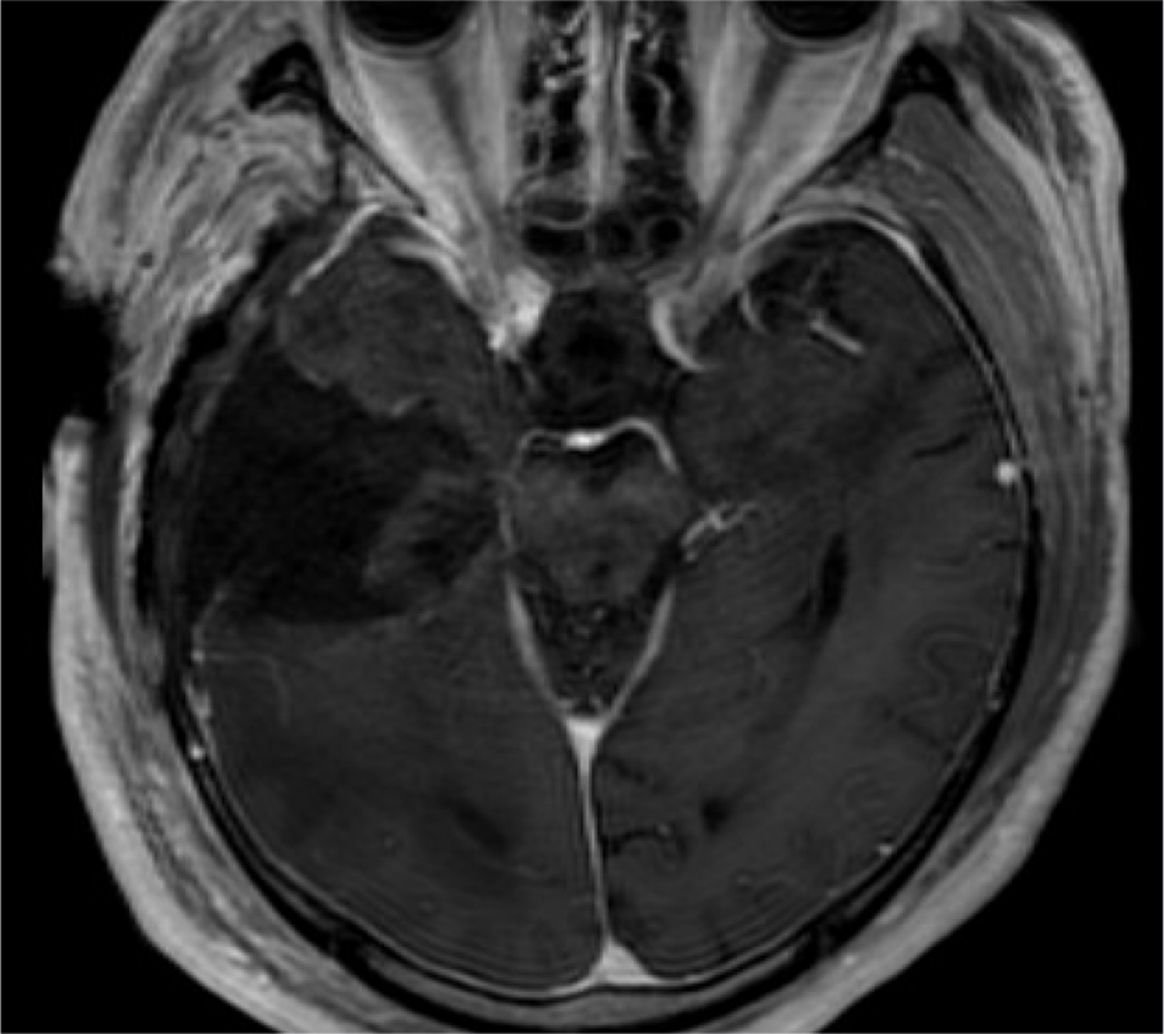
Contrast-enhanced *T*_1_ weighted image performed the day after the surgery reveals no residual tumour.

Complete surgical resection was achieved and no residual tumour was evident on the postoperative MRI (Figure 5). No surgical complications occurred. Although the patient experienced more drowsiness than before, and lost his appetite after the operation, he gradually recovered and was discharged. On the basis of the histopathological diagnosis, three-dimensional conformal radiation therapy with a dose of 61.2 Gy in 34 fractions and chemotherapy with temozolomide were administered. At the 9-month follow-up, the MRI revealed multiple vertebral metastases. The patient died 13 months after the surgery.

## Discussion

Primary angiosarcoma of the CNS is extremely rare, with only 31 cases reported in the literature.^1,2,4–7^ Males (65%) were more frequently affected than females (35%).^6^ Most angiosarcomas of the CNS occur in the brain parenchyma and a few in the meninges. Patients range in age from neonates to the elderly. There is, usually, a rapid onset of neurological symptoms that relate to the tumour’s location.

Although the aetiology of CNS sarcoma remains unknown, various possible aetiologies have been proposed, such as previous radiation therapy.^8^ Radiation-induced angiosarcoma of the brain was reported in a single case of Thorotrast-associated angiosarcoma.^2^ Thorotrast, which was used as a contrast medium in the 1930s and 1940s, contains particles of the radioactive compound thorium dioxide.^2^ To the best of our knowledge, an angiosarcoma of the brain arising after external beam radiation therapy has not been reported.

Imaging studies of CNS angiosarcoma have demonstrated a haemorrhagic, well-demarcated lesion with avid contrast enhancement and significant vasogenic oedema.^4^ The CT scan and MRI findings of our case too were similar to those reported previously.^4^ Although angiographic findings of CNS angiosarcoma have not been reported previously, hepatic angiosarcoma was reported to show a peripheral tumour stain, and the appearance of puddling during the mid-arterial phase that persisted for as long as 34 s.^9^ A peripheral enhancement was not obvious in our case, but contrast medium pooling, similar to puddling, was observed. The differential diagnosis of an angiosarcoma of the brain includes assessments for cavernous malformation,^7^ glioblastoma and metastasis. Particularly, cavernous malformation and glioblastoma, similar to angiosarcoma, can also arise secondarily to radiation therapy.^10,11^ An angiosarcoma is thought to be more progressive and more strongly enhanced than a cavernous malformation. It may have more demarcated margins compared with a glioblastoma. Brain metastasis is determined based on clinical history and further careful exploration of the whole body.

While the primary therapy for CNS angiosarcoma is surgical resection, a few patients have benefited from radiation therapy and/or chemotherapy too. Although chemotherapy for angiosarcoma in the CNS is not established, the use of temozolomide, doxorubicin, paclitaxel and bevacizumab, or their combination, has been reported.^5^

An angiosarcoma of the brain is characterized by a high rate of local recurrence and a short median survival time. In one series of seven patients, four died within 4 months of surgery and one after 30 months.^1^ The remaining two young patients were alive at 39 and 102 months after surgery.

Although an accurate preoperative diagnosis of an angiosarcoma of the brain is difficult, radiologists and neurosurgeons need to be aware that it may occur long after external beam radiotherapy.

## Learning points

1. Angiosarcoma of the brain may occur long after external beam radiotherapy.
2. Angiosarcoma of the brain shows a haemorrhagic mass with avid contrast enhancement and significant vasogenic oedema.
3. Contrast medium pooling on angiography may be a characteristic finding of angiosarcoma.
